# Weekend physical activity profiles and their relationship with quality of life: The SOPHYA cohort of Swiss children and adolescents

**DOI:** 10.1371/journal.pone.0298890

**Published:** 2024-05-31

**Authors:** Ranin Darkhawaja, Johanna Hänggi, Bettina Bringolf-Isler, Bengt Kayser, L. Suzanne Suggs, Marek Kwiatkowski, Nicole Probst-Hensch

**Affiliations:** 1 Swiss Tropical and Public Health Institute, Allschwil, Switzerland; 2 University of Basel, Basel, Switzerland; 3 Institute of Sport Sciences, University of Lausanne, Lausanne, Switzerland; 4 Institute for Public Health and Institute of Communication and Public Policy, Università della SvizzeraItaliana, Lugano, Switzerland; Hamasaki Clinic, JAPAN

## Abstract

**Introduction:**

Quality of life (QoL) is an important health indicator among children and adolescents. Evidence on the effect of physical activity (PA)-related behaviors on QoL among youth remains inconsistent. Conventional accelerometer-derived PA metrics and guidelines with a focus on whole weeks may not adequately characterize QoL relevant PA behavior.

**Objective:**

This study aims to a) identify clusters of accelerometer-derived PA profiles during weekend days among children and adolescents living in Switzerland, b) assess their cross-sectional and predictive association with overall QoL and its dimensions, and c) investigate whether the associations of QoL with the newly identified clusters persist upon adjustment for the commonly used PA metrics moderate-to-vigorous physical activity (MVPA) and time spent in sedentary behavior (SB).

**Methods:**

The population-based Swiss children’s Objectively measured PHYsical Activity (SOPHYA) cohort among children and adolescents aged 6 to 16 years was initiated at baseline in 2013. PA and QoL information was obtained twice over a five-year follow-up period. The primary endpoint is the overall QoL score and its six dimension scores obtained by KINDL® questionnaire. The primary predictor is the cluster membership of accelerometer-derived weekend PA profile. Clusters were obtained by applying the k-medoid algorithm to the distance matrix of profiles obtained by pairwise alignments of PA time series using the Dynamic Time Warping (DTW) algorithm. Secondary predictors are accelerometer-derived conventional PA metrics MVPA and SB from two combined weekend days. Linear regression models were applied to assess a) the cross-sectional association between PA cluster membership and QoL at baseline and b) the predictive association between PA cluster membership at baseline and QoL at follow-up, adjusting for baseline QoL.

**Results:**

The study sample for deriving PA profile clusters consisted of 51.4% girls and had an average age of 10.9 [SD 2.5] years). The elbow and silhouette methods indicated that weekend PA profiles are best classified in two or four clusters. The most differentiating characteristic for the two-clusters classification (“lower activity” and “high activity”), and the four-clusters classification (“inactive”, “low activity”, “medium activity”, and “high activity”), respectively was the participant’s mean counts per 15-seconds epoch. Participants assigned to high activity clusters were younger and more often male. Neither the clustered PA profiles nor MVPA or SB were cross-sectionally or predictively associated with overall QoL. The only association of a conventional PA metrics with QoL while adjusting for cluster membership was observed between MVPA during the weekend days and social well-being with a mean score difference of 2.4 (95%CI: 0.3 to 4.5; p = 0.025).

**Conclusion:**

The absence of strong associations of PA metrics for the weekend with QoL, except for the positive association between MVPA during the weekend days and social well-being, is in line with results from two randomized studies not showing efficacy of PA interventions on youth QoL. But because PA decreases with age, its promotion and relevance to QoL remain important research topics. Larger longitudinal study samples with more than two follow-up time points of children and adolescents are needed to derive new novel accelerometer-derived PA profiles and to associate them with QoL dimensions.

## Introduction

Quality of life (QoL) is an important health determinant and indicator, which complements other conventional health indicators such as mortality and morbidity. The World Health Organization (WHO) defines QoL as “an individual’s perception of their position in life in the context of the culture and value systems in which they live and in relation to their goals, expectations, standards and concerns”. QoL is a subjective and multidimensional well-being construct that includes physiological, psychological, and functional aspects [1].

According to the WHO Mental Health Division, the measurement of QoL among children and adolescents should be age-appropriate, applicable independent of the health status of the targeted group, appropriate cross-culturally, and include both positive and negative aspects. There is a preference for self-reported measurement. QoL assessment should consider aspects of health, subjective well-being and social indicators [2]. The KINDL® questionnaire is an accepted, valid and reliable QoL instrument for children and adolescents [3].

Few studies assessed QoL in youth in a population-representative manner. High QoL in children and adolescents is essential for a healthy transition to adulthood and for maintaining a good QoL later in life, in line with the United Nations Sustainable Development Goal 3 of ensuring good health and improving QoL for all [4]. Furthermore, the understanding of health and well-being before adulthood is relevant in itself, in line with Article 12 of the United Nation’s Convention of the Rights of the Child [5].

Population-based assessment of QoL and its determinants in the young is important for evaluating the impact of existing public health programs and policies targeting children and adolescents [6]. In recent years, there has been a growing focus on understanding the relationship of physical activity (PA) and sedentary behavior (SB) with the overall QoL and its dimensions among children and adolescents without chronic health conditions [7, 8], beyond the well-established evidence for the effect of PA and SB on physical health [9, 10]. In these studies of children sampled from the general population, inconsistencies and the absence of associations between PA-related behaviors and QoL exist. They may in part reflect challenges in measuring, characterizing, and summarizing PA and SB as relevant for specific health and well-being endpoints.

PA related behavior is measured by applying subjective [11] and objective [12, 13] methods. PA questionnaires have the advantage of being low cost, easily applicable and having highly acceptable rate among the participants [14], but they are not valid for measuring overall PA in youth [15–20]. Accelerometers are the most commonly used instruments for objectively measuring PA-related behaviors [12, 21] and allow characterizing it in different dimensions [22, 23].

Dimensions of PA-related behavior include Frequency, Intensity, Time, and Type (FITT) [24]. Different dimensions of PA-related behaviors have specific and in part independent health benefits [25, 26]. Even low levels of PA can have health benefits [27–29]. Moving from an inactive to an active state promotes health considerably [27, 30]. Moreover, with regard to time, children’s and adolescents’ PA and SB vary between weekdays and weekend days. The young tend to be more physically active on weekdays compared to weekend days [31, 32]. They are generally more likely to engage in unhealthy behaviors including SB during weekend days [33].

Many studies on PA-related behaviors and health or QoL are still questionnaire-based and focus on established PA metrics such as time in a week spent in SB or in moderate-to-vigorous physical activity (MVPA) [34]. These categories also form the basis of national and international PA recommendations to date [30]. Yet, they are unlikely to capture the entire health and well-being relevant heterogeneity of PA and PA-related behaviors between individuals [34]. The rich data captured by activity sensors such as accelerometers contain additional information with the potential to unlock novel insights into the association of specific PA-patterns with health endpoints including QoL [35, 36]. The pattern difference in the accumulation of certain PA-related behavior over time can have significant implication [37]. Dynamic time warping (DTW) enabled progress in the more exhaustive utilization of sensor based time series data such as, captured by the accelerometer. It is a technique proved appropriate and unique for measuring cross-correlated differences between sensor based time series data sets from two aspects; first, the difference in time traces and second, the difference in the motion paths taken [38].

This study aimed to a) identify clusters of accelerometer-derived PA profiles during weekend days among children and adolescents from the Swiss children’s Objectively measured PHYsical Activity (SOPHYA) cohort, b) assess their cross-sectional and predictive association with overall QoL and its dimensions, and c) investigate whether QoL associations with newly identified clusters persist upon adjustment for established PA indicators.

## Methods

### Study design and population

The present study was conducted among children and adolescents participating in the baseline assessment of the SOPHYA cohort (SOPHYA1) between November 19, 2013 and May 28, 2015 [39, 40]. All youth who were registered in Switzerland and born between 1998 and 2007 were eligible. The Federal Statistical Office drew random samples from this sampling frame stratified by sex, year of birth, and language (German; French; Italian). The recruitment and the participation rate in SOPHYA1 was described before [39, 40]. In short, the participation rate among 2032 families who answered to the SOPHYA1 baseline interview was 65%. Valid accelerometer measurements accompanied by self-administrated questionnaires during the measurement week were obtained from 1320 youth aged 6 to 16 years. The SOPHYA1 baseline accelerometry formed the basis for deriving PA profiles and for the assessment of the cross-sectional association between clusters of PA profiles and QoL.

For the assessment of the predictive association of clusters of PA profiles at baseline, QoL data obtained at the follow-up assessment between January 9, 2019 and November 20, 2020 (SOPHYA2 accelerometry) was considered as outcome. SOPHYA2 was based on the 1,320 SOPHYA1 baseline accelerometry participants who provided self-administered questionnaire information on socio-demographic characteristics, weight, height, and QoL. Of these participants, 844 could be re-contacted by phone in 2019 and 780 of them provided consent to be re-contacted for a follow-up accelerometer measurement. Among them, 447 participants finally had valid accelerometer measurements as well as self-reported socio-demographic characteristics, weight, height, and QoL.

In SOPHYA1, a parent gave written informed consent (IC) for their children’s participation. Adolescents aged 12 years or older filled in an additional IC form. In SOPHYA2, for participants younger than 14 years written IC was provided by a parent as proxy; for participants aged between 14 and 18 years, both parental and an own written IC was provided; for youth above 18 years only own written IC was given.

### Data collection

Since participants were spread across Switzerland, contact with them was exclusively remote. The regional SOPHYA-study partners (German-speaking region: Swiss Tropical and Public Health Institute in Basel; French-speaking regions: University of Lausanne; Italian-speaking regions: Università della Svizzera Italiana) coordinated participant assessment.

1. **Telephone interview.** At baseline and follow-up as a first SOPHYA assessment computer-assisted telephone interviews in the respective language region (German; French; Italian) were conducted with one parent as proxy for all children (SOPHYA1), and with children 15 years or older or with one parent as proxy for children aged 14 years or younger (SOPHYA2), respectively. Interview data collected included sociodemographic characteristics (sex, language region (based on the zip code), nationality, urbanicity (based on the zip code), parental education, and household income).
2. **Accelerometer measurement.** At baseline and follow-up, instructions were given via phone to families on how to use the accelerometer. Subsequently, an accelerometer and written instructions were mailed to the address of the participants with a pre-paid postage box to return the devices to the investigators after completion of the measurements. Most of the participants wore Actigraph accelerometer model GT3X, while few of them wore GT1M, (ActiGraph, Pensacola, Florida, USA), both producing comparable output [41–43]. The device was tied to the participant’s right hip with an elastic band and worn for seven consecutive days except when the participant was performing water activities or was sleeping. To ensure the detection of shorter bursts of PA, which are typical for children [44], the device was set without filtering and in 15-seconds epoch mode (measured as milli-gravity units, mg). ActiLife 6.2 software (ActiGraph, Pensacola, Florida, USA) was used to initialize the device, to download the data and to process the data. Non-wearing time was defined as any period of 60 or more minutes of consecutive zero counts.
3. **Paper-based survey.** At SOPHYA1 and SOPHYA2, families participating in the accelerometry sub-study received an additional paper-based survey to answer questions on the child’s age when the accelerometer measurements took place, sport behavior during the measured week, their weight, their height and any diagnosis of chronic disease. Additionally, the survey included the validated KINDL® questionnaire for assessing children’s QoL. The questionnaire was administered in the three language areas in Switzerland using the official translation of the questionnaire (Romansh-speaking people filled in the German questionnaire). Validated questionnaire versions tailored to different age groups are available for self-assessment and as parent-proxy tool [45, 46]. In SOPHYA1, the questionnaire was filled out by a parent. At follow-up in SOPHYA2, the participants themselves completed the questionnaires given their higher age.

### Statistical analysis

#### 1. Study sample

The study samples for this current paper are described in **S1 and S2 Figs**. Based on the subsequent inclusion and exclusion criteria, the sample size for deriving clusters of PA profiles at baseline and for the cross-sectional association of PA profiles with QoL at baseline was N = 926, and the sample size for the predictive association of clusters of PA profiles at baseline with QoL at follow-up was N = 292.

Inclusion criteria

For deriving clusters of PA profiles on the weekend days at baseline (where the sample was much larger), and for associating clusters of PA profiles cross-sectionally in SOPHYA1 with QoL the following inclusion criteria was applied:

- Participants were restricted to those providing valid accelerometry baseline data from at least 8 hours of wear time for one Saturday and one Sunday, respectively, with both days from the same weekend. In the cases where accelerometers were worn for two consecutive weekends or more, the first weekend was chosen.
- Participants were required to have complete SOPHYA1 data for the overall QoL and its dimensions (see below for partially missing information) and for the selected covariates (age, sex, parental education, household income, language region, nationality, urbanicity, self-reported diagnosis with at least one chronic disease and season of measurement).
- Additional criteria for associating clusters of PA profile predictively with QoL at follow-up:
- Participants were additionally required to have complete data for overall QoL and its dimensions at follow-up (see below for partially missing information).

Exclusion criteria (for all analyses)

- Participants self-reporting a diagnosis of epilepsy or arthropathy at either SOPHYA1 or SOPHYA2.
- Participants with accelerometer data collected at 60-seconds epoch time.
- Participants lacking acceleration data in all three axes.

#### 2. Measures

Primary endpoint: QoL

The validated KINDL® QoL questionnaire consists of 24 items, each answered on a five-point ordinal Likert scale ranging from “never” ( = 5) to “always” ( = 1). Each item belongs to one of the six QoL dimensions (four items per dimension): physical well-being, emotional well-being, self-esteem, family connection, social well-being and functioning at school. The QoL dimensions are scored separately as the sum of the scores of 4 items, ranging from 4 to 20. The domain specific scores are subsequently transformed to a scale from 0 to 100. The overall QoL score is calculated based on the mean value of all answered items (https://www.kindl.org/english/analysis/). Higher scores represent a higher QoL. If missing values occurred and affected less than 70% of the answers contributing to a dimension or the total score, the algorithm proposed by the authors of the KINDL® questionnaire was used to replace these missing data [47]. If more than 70% of the answers were missing, the score of the respective participant was excluded from the analysis. Based on this, the exclusion criteria affected 2% of the participants in SOPHYA1 and less than 1% participants in SOPHYA2 [48].

Main predictors

a. Primary predictor: PA profile cluster membership
  Every participant was assigned to a cluster based on their accelerometer-recorded weekend PA profile (See section **“Statistical analysis steps: In a second step”** for details), and the cluster membership indicator variables were used as the main explanatory variables in the subsequent statistical analyses.
b. Secondary predictors: Established physical activity metrics
  - Average MVPA in hours per day during the weekend
  - The average MVPA in hours per day during the weekend was derived by ActiLife 6.2, which is based on the age-dependent cut-offs of Freedson [49] with a threshold of four metabolic equivalents [50].
  - Average SB in hours per day during the weekend
  - The average SB in hours per day during the weekend was derived by ActiLife 6.2, which is defined as an intensity of less than 100 cpm [51].

Covariates

a) Sociodemographic characteristics
  Age; sex (boy, girl); language region (German, French, Italian); nationality (Swiss, foreign nationality, Swiss dual citizen); urbanicity (agglomeration, rural, urban); participation in organized sport activities (child participate in sport club at least once a week, child does not participate in a sport club at least once a week); parental education (apprenticeship, high school diploma, higher vocational training, undefined category, compulsory school, diploma school, not willing to provide information); and monthly household income (≤ 6,000 CHF, 6,001 to 9,000 CHF, 9,000 more CHF, not willing to provide information, missing).
b) Health indicator
  Self-reported diagnosis of at least one chronic disease (did not have any of the chronic diseases, had at least one chronic disease).
c) Use of the accelerometer
  Season of measurement (spring, summer, autumn, winter).

#### 3. Statistical analysis steps

In a first step, we calculated descriptive statistics (n, %, mean, SD) for characterizing the study populations included in cross-sectional and predictive analyses at baseline **Table 1**

In a second step, we clustered profiles of accelerometer-derived PA from two combined weekend days using the k-medoid algorithm on a distance matrix obtained by DTW [52]. DTW calculates normalized pairwise dissimilarity score of time series and has previously been applied to study accelerometer data [53, 54]. Using the DTW distance matrix, the k-medoid algorithm [55] finds “k” representative participants, called medoids, by minimizing the average DTW dissimilarity of all the participants’ PA profiles to the nearest candidate medoid. Then, each participant is assigned to the cluster represented by the nearest medoid [56, 57]. This algorithm requires choosing the number of clusters (k) in advance. We have applied the widely used elbow and the silhouette methods to select the optimal number of clusters. In the main manuscript we present the results for k=4. Supplementary materials contain analogous data for k=2. All calculations were performed in R v4.2.1 [58], using the dtw v1.23-1 [52] for the DTW algorithm and cluster v2.1.2 [55] for clustering. Calculations were performed at sciCORE (http://scicore.unibas.ch/) scientific center at University of Basel.

In a third step, we described the distribution within the derived clusters of sociodemographic characteristics, health indicators including QoL, established PA metrics **(Table 2 and S2 Table)** and several ad-hoc PA profile summaries (**Fig 3** and **S4 Fig**).

In a fourth step, the cross-sectional association between cluster membership and QoL scores (overall and dimensions) at baseline without (Model 1) and with adjusting for established PA metrics (Model 2: adjusting for MVPA; Model 3: adjusting for SB) was estimated with linear regression models. All models were adjusted for age, sex, language region, nationality, urbanicity, participation in organized sport activities, household income, parental education, self-reported diagnosis with at least one chronic disease, and season of measurement (**Tables 3–5** for 4 clusters and **S3–S5 Tables** for 2 clusters).

In a fifth step, the predictive association between cluster membership at baseline and QoL scores (overall; dimensions) at follow-up was estimated in the same manner as in step four, with additional adjustment for QoL at baseline. The sample was restricted to participants in SOPHYA2 accelerometry (**S6–S11 Tables** for 4 clusters and for 2 clusters).

## Results

### Characteristics of the study participants

Baseline characteristics of the study population are summarized in **Table 1**. The SOPHYA1 study sample for the deriving the clusters of PA profiles and for assessing the cross-sectional QoL association consisted of 926 children and adolescents (48.6% boys, 51.4% girls). The average age of the participants was (mean [SD]: 10.9 [2.5] years). The majority of the participants were of Swiss nationality (68.7%) from the German-speaking region (71.3%) reflecting Swiss demographics. The average overall QoL score was (mean [SD]: 81.1 [8.3] points). Of the specific QoL dimensions, self-esteem had the lowest score (mean [SD]: 75.7 [13.7] points), while emotional well-being exhibited the largest score (mean [SD]: 86.4 [10.7] points). The mean of time spent in MVPA and in SB were (mean [SD]: 1.1 [0.7] hr/day) and (mean [SD]: 7.6 [1.6] hr/day), respectively.

**Table 1:** Characteristics of study participants at baseline (SOPHYA1; 2013) assessment.

N=926	
Sociodemographic characteristics	Mean (SD) / N (%)
**Age**	10.9 (2.5)
**Sex**	
• *Boy*	450.0 (48.6%)
• *Girl*	476.0 (51.4%)
**Language region**	
• *German*	660.0 (71.3%)
• *French*	174.0 (18.8%)
• *Italian*	92.0 (9.9%)
**Nationality**	
• *Swiss*	636.0 (68.7%)
• *Foreign nationality*	95.0 (10.3%)
• *Swiss dual citizen (Swiss and foreign nationality)*	195.0 (21.1%)
**Urbanicity**	
• *Agglomeration*	438.0 (47.3%)
• *Rural*	305.0 (32.9%)
• *Urban*	183.0 (19.8%)
**Parental education** Highest parental education	
• *Apprenticeship*	409.0 (44.2%)
• *High school diploma*	214.0 (23.1%)
• *Higher vocational training*	168.0 (18.1%)
• *Undefined category*	84.0 (9.1%)
• *Compulsory school*	34.0 (3.7%)
• *Diploma school*	16.0 (1.7%)
• *Not willing to provide information*	1.0 (0.1%)
**Household income**	
• *≤ 6*,*000 CHF*	195.0 (21.1%)
• *6*,*001 to 9*,*000 CHF*	299.0 (32.3%)
• *9*,*000 and more CHF*	334.0 (36.1%)
• *Not willing to provide information*	31.0 (3.3%)
• *Missing*	67.0 (7.2%)
**Health indicators**	**Mean (SD) / n (%)**
**Self-reported diagnosis with at least one chronic disease**The participant self-reported at least one of the following chronic diseases: asthma, hay fever, allergy, atopic dermatitis, diabetes mellitus, chronic enteritis, hypertension, epilepsy, and arthropathy and attention deficit hyperactivity disorder. Or any other chronic disease not specifically included in the mentioned list	
• *Did not have any of the chronic diseases*	636.0 (68.7%)
• *Had at least one chronic disease*	290.0 (31.3%)
**Quality of life**	
• *Overall QoL*	81.1 (8.3)
• *Physical well-being*	84.3 (12.9)
• *Emotional well-being*	86.4 (10.7)
• *Self-esteem*	75.7 (13.7)
• *Family connection*	81.6 (12.5)
• *Social well-being*	78.3 (12.5)
• *Functioning at school*	80.4 (14.7)
**Use of the accelerometer**	**Mean (SD) / n (%)**
**Season of measurement**	
• *Spring*	269.0 (29.0%)
• *Summer*	128.0 (13.8%)
• *Autumn*	222.0 (24%)
• *Winter*	307.0 (33.2%)
**Conventional physical activity measures during the weekend**	**Mean (SD)**
**Sedentary Behavior during weekend days** Derived by ActiLife 6.2, which is defined as an intensity of less than 100 cpm	
• *Average time in sedentary behavior (hours/day)*	7.6 (1.6)
**Moderate to Vigorous Physical Activity during weekend days** Derived by ActiLife 6.2, which is based on the age-dependent cut-offs of Freedson with a threshold of four metabolic equivalents	
• *Average time in moderate to vigorous physical activity (hours/day)*	1.1 (0.7)
**Mean counts per epoch on weekend days** Average of the total physical activity counts based on the vector magnitude axis	143.9 (58.6)

Baseline characteristics comparing participants included versus not included in the predictive association analysis are presented in **S1 Table**. The sample size for assessing the predictive associations was 292. Youth participating at follow-up tended to be younger and more active and to have higher QoL scores at baseline.

### Clusters of PA profiles

**Fig 1:**
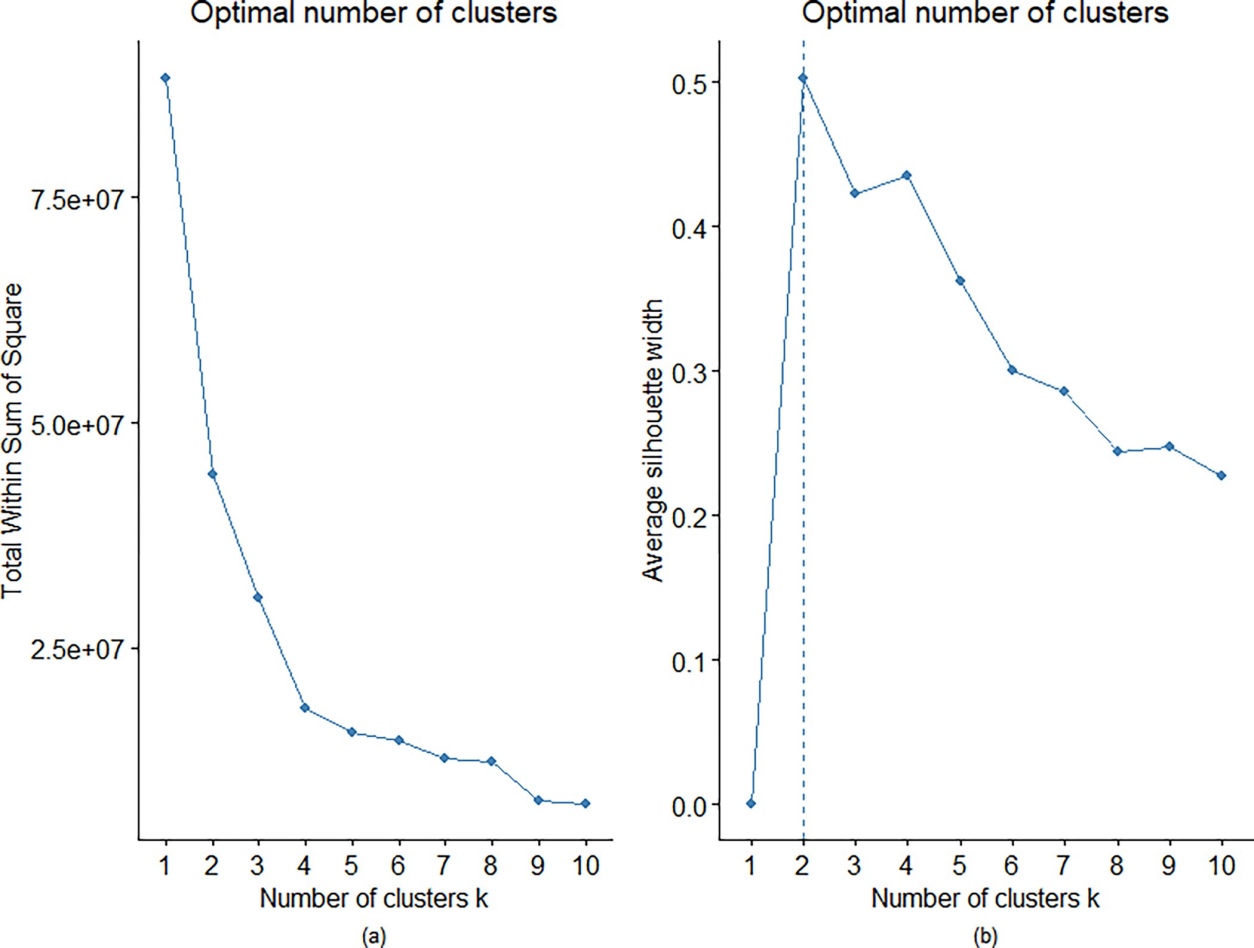
Number of physical activity profile clusters using (a) Elbow and (b) Silhouette methods.

The elbow and the silhouette methods were applied to determine the optimal number of clusters k **(Fig 1)**. The elbow plot **(Fig 1A)** points to a substantial reduction in the within-cluster dis-similarity when moving from one to two and then, more noticeably, three to four clusters. The silhouette plot **(Fig 1B)** suggests that PA profiles are best described with two clusters, but the four-cluster model scores highly as well. Therefore, we conducted parallel analyses with k=4 (main text) and k=2 (Online Supplement).

**Fig 2:**
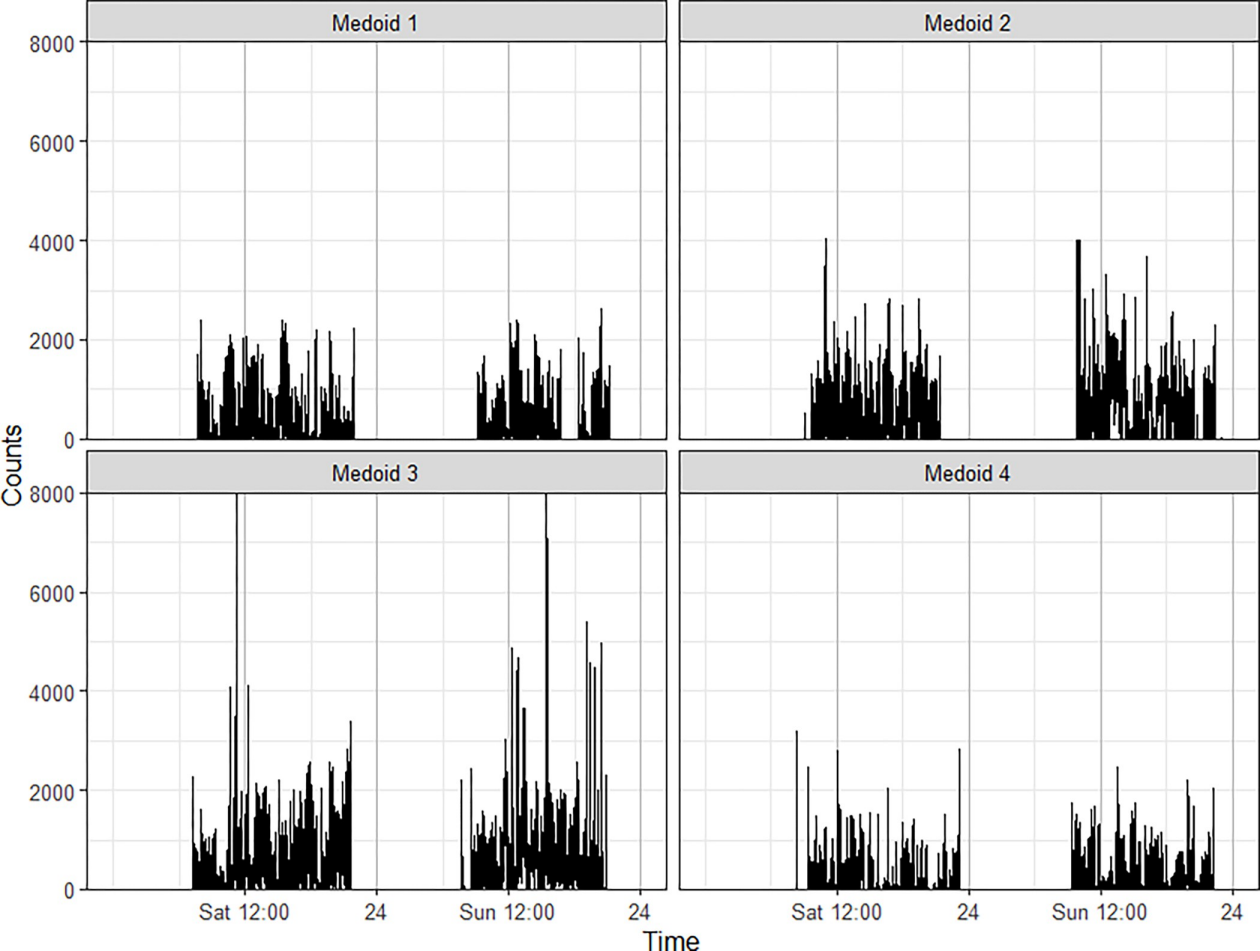
Physical activity pattern (counts in 15-seconds epoch) of the clusters’ four medoids (participants).

**Fig 3:**
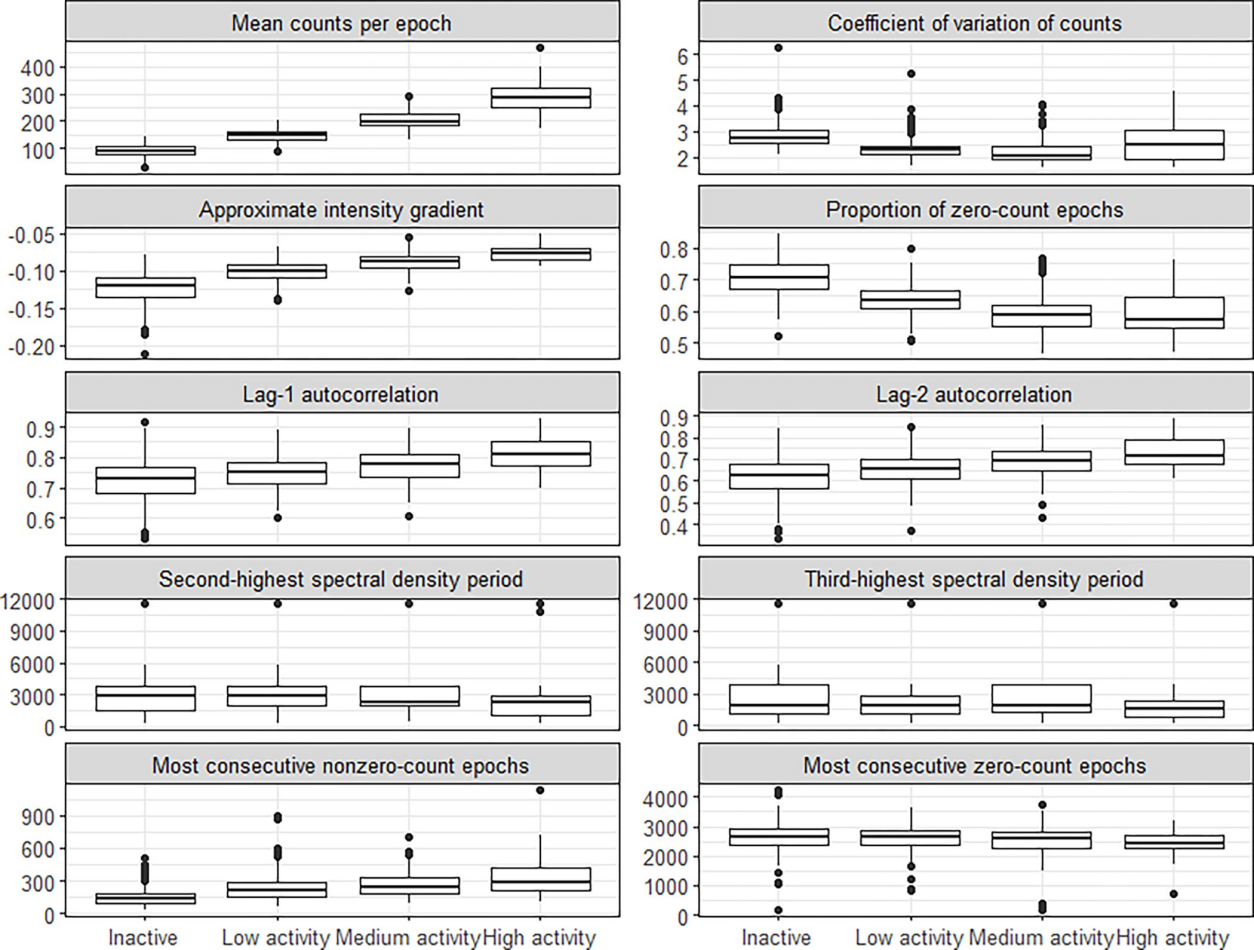
Distributions of summaries of physical activity patterns per cluster.

The four clusters differed markedly in the participants’ overall level of PA as measured by mean counts per 15-seconds epoch **(Figs 2 and 3)**. Accordingly, they were labeled as “inactive”, “low activity”, “medium activity”, and “high activity” clusters. We calculated additional summaries of the count time series to identify qualitative differences between clusters beyond average activity levels. These metrics were: autocorrelation at lag-1 (15 seconds) and lag-2 (30 seconds) (correlation between values that are 15 seconds and 30 seconds apart, respectively); coefficient of variation (standard deviation of epoch counts divided by the mean); approximate intensity gradient (slope of linear regression of log counts on log number of epochs with that number of counts) [59]; the time periods with second and third highest spectral density (the highest always corresponding to 24h due to the diurnal cycle); the longest number of consecutive epochs with non-zero and with zero counts; and the proportion of zero-count epochs). The distribution of these summaries in each cluster visualized in **Fig 3** differ notably between clusters for the autocorrelation, intensity gradient and proportion of zero-count epochs.

### Distribution of participant characteristics according to clusters of PA profiles

The distribution of sociodemographic characteristics, health indicators, including QoL, accelerometry use, and conventional PA metrics between clusters of PA profiles are presented in **Table 2**. The “high activity” cluster participants, compared to the other clusters, scored higher in the mean counts per epoch (mean [SD]: high activity: 284.0 [61.0]; medium activity: 205.0 [29.6]; low activity: 148.0 [19.3]; inactive: 90.9 [21.1] counts; p <0.001). Average sedentary time was highest in the “inactive” cluster (mean [SD]: high activity: 6.3 [1.4]; medium activity: 6.6 [1.3]; low activity: 7.2 [1.3]; inactive: 8.5 [1.5] hr/day; p<0.001) and average hours spent in MVPA was highest in the “high activity” cluster’s participants (mean [SD]: high activity: 2.4 [0.9]; medium activity: 1.8 [0.6]; low activity: 1.2 [0.4]; inactive: 0.6 [0.3] hr/day; p<0.001). The higher activity cluster participants were on average younger (mean [SD]: high activity: 10.2 [2.3]; medium activity: 9.7 [2.1]; low activity: 10.2 [2.1]; inactive: 12.4 [2.4] years; p<0.001) and with an overrepresentation of the male gender (p<0.001) with the percentages of boys in the “high activity”, “medium activity”, “low activity” and “inactive” clusters 66.7%, 60.8%, 48.9%, and 39.5%, respectively. There was a tendency for QoL overall and its dimensions to go from being highest in the “high activity” cluster to lowest in the “inactive” cluster’s participants. This reached statistical significance for overall QoL (p<0.001), physical well-being (p<0.001) and functioning at school (p<0.001).

**Table 2:** Characteristics of study participants at baseline (SOPHYA; 2013), by cluster of physical activity profile.

N = 926					
	High activity n = 39 (4.2%)	Medium activity n = 209 (22.6%)	Low activity n = 313 (33.8%)	Inactive n = 365 (39.4%)	P-value
Variable	Mean (SD)/ n (%)	Mean (SD)/ n (%)	Mean (SD)/ n (%)	Mean (SD)/ n (%)	
**Socio-demographic characteristics**					
**Age**	10.2 (2.3)	9.7 (2.1)	10.2 (2.1)	12.4 (2.4)	<0.001 P-value from the analysis of variance (ANOVA)
**Sex**					
• *Boy*	26.0 (66.7%)	127.0 (60.8%)	153.0 (48.9%)	144.0 (39.5%)	<0.001 P-value from the chi-squared test
• *Girl*	13.0 (33.3%)	82.0 (39.2%)	160.0 (51.1%)	221.0 (60.5%)	
**Language region**					
• *German*	29.0 (74.4%)	155.0 (74.2%)	230.0 (73.5%)	246.0 (67.4%)	0.202 P-value from Finsher’s exact test
• *French*	9.0 (23.1%)	34.0 (16.3%)	58.0 (18.5%)	73.0 (20.0%)	
• *Italian*	1.0 (2.6%)	20.0 (9.6%)	25.0 (8.0%)	46.0 (12.6%)	
**Nationality**					
• *Swiss*	27.0 (69.2%)	148.0 (70.8%)	218.0 (69.6%)	243.0 (66.6%)	0.4423
• *Foreign nationality*	3.0 (7.7%)	19.0 (9.1%)	39.0 (12.5%)	34.0 (9.3%)	
• *Swiss dual citizen (Swiss and foreign nationality)*	9.0 (23.1%)	42.0 (20.1%)	56.0 (17.9%)	88.0 (24.1%)	
**Urbanicity**					
• *Agglomeration*	14.0 (35.9%)	86.0 (41.1%)	143.0 (45.7%)	195.0 (53.4%)	0.4423
• *Rural*	13.0 (33.3%)	79.0 (37.8%)	105.0 (33.5%)	108.0 (29.6%)	
• *Urban*	12.0 (30.8%)	44.0 (21.1%)	65.0 (20.8%)	62.0 (17.0%)	
**Parental education**					
• *Apprenticeship*	17.0 (43.6%)	89.0 (42.6%)	134.0 (42.8%)	169.0 (46.3%)	0.6033
• *High school diploma*	11.0 (28.2%)	45.0 (21.5%)	66.0 (21.1%)	92.0 (25.2%)	
• *Higher vocational training*	8.0 (20.5%)	39.0 (18.7%)	59.0 (18.8%)	62.0 (17.0%)	
• *Undefined category*	2.0 (5.1%)	27.0 (12.9%)	33.0 (10.5%)	22.0 (6.0%)	
• *Compulsory school*	1.0 (2.6%)	6.0 (2.9%)	14.0 (4.5%)	13.0 (3.6%)	
• *Diploma school*	0.0 (0.0%)	3.0 (1.4%)	6.0 (1.9%)	7.0 (1.9%)	
• *Not willing to provide information*	0.0 (0.0%)	0.0 (0.0%)	1.0 (0.3%)	0.0 (0.0%)	
**Household income**					
• *≤ 6*,*000 CHF*	8.0 (20.5%)	36.0 (17.2)	62.0 (19.8%)	89.0 (24.4%)	0.1643
• *6*,*001 to 9*,*000 CHF*	14.0 (35.9%)	62.0 (29.7%)	98.0 (31.3%)	125.0 (34.2%)	
• *9*,*000 and more CHF*	14.0 (35.9%)	83.0 (39.7%)	126.0 (40.3%)	111.0 (30.4%)	
• *Not willing to provide information*	1.0 (2.6%)	10.0 (4.8%)	11.0 (3.5%)	9.0 (2.5%)	
• *Missing*	2.0 (5.1%)	18.0 (8.6%)	16.0 (5.1%)	31.0 (8.5%)	
**Health indicators**					
**Self-reported diagnosis with at least one chronic disease**					
• *Did not have any of the chronic diseases*	30.0 (76.9%)	155.0 (74.2%)	211.0 (67.4%)	240.0 (65.8%)	0.1233
• *Had at least one chronic disease*	9.0 (23.1%)	54.0 (25.8%)	102.0 (32.6%)	125.0 (34.2%)	
**Quality of life**					
• *Overall QoL*	83.2 (6.6)	82.6 (7.5)	81.6 (8.3)	79.6 (8.7)	<0.0011
• *Physical well-being*	85.7 (10.6)	88.1 (10.3)	84.9 (13.0)	81.4 (13.8)	<0.0011
• *Emotional well-being*	88.1 (9.2)	87.2 (9.7)	86.6 (11.2)	85.6 (11.0)	0.1731
• *Self-esteem*	76.3 (14.8)	76.2 (13.2)	76.4 (13.5)	74.7 (13.9)	0.0891
• *Family connection*	82.4 (10.7)	81.8 (11.3)	80.7 (12.9)	82.2 (13.0)	0.1381
• *Social well-being*	82.5 (11.7)	78.8 (11.4)	78.4 (11.6)	77.5 (13.8)	0.3781
• *Functioning at school*	84.0 (11.9)	83.2 (13.9)	82.7 (13.8)	76.5 (15.4)	<0.0011
**Use of the accelerometer**					
**Season of measurement**					
• *Spring*	13.0 (33.3%)	54.0 (25.8%)	86.0 (27.5%)	116.0 (31.8%)	<0.0013
• *Summer*	7.0 (17.9%)	42.0 (20.1%)	43.0 (13.7%)	36.0 (9.9%)	
• *Autumn*	15.0 (38.5%)	58.0 (27.8%)	76.0 (24.3%)	73.0 (20.0%)	
• *Winter*	4.0 (10.3%)	55.0 (26.3%)	108 (34.5%)	140.0 (38.4%)	
**Conventional physical activity measures during the weekend**					
**Sedentary Behavior on weekend days**					
• *Average time in sedentary in hours/day*	6.3 (1.4)	6.6 (1.3)	7.2 (1.3)	8.5 (1.5)	<0.0011
**Moderate to Vigorous Physical Activity on weekend days**					
• *Average time in moderate to vigorous physical activity in hours/day*	2.4 (0.9)	1.8 (0.6)	1.2 (0.4)	0.6 (0.3)	<0.0011
**Mean counts per epoch on weekend days**	284.0 (61.0)	205.0 (29.6)	148.0 (19.3)	90.9 (21.1)	<0.0011

### Cross-sectional association of clusters of PA profiles with QoL

The association of cluster membership with QoL was first estimated without adjustment for conventional PA metrics **(Model 1, Table 3)**. No statistically significant differences between the reference “inactive” cluster and the remaining three were present, albeit a suggestion for a positive trend for increasing QoL with increasing activity was observable. The strongest trend for an increasingly positive association of more activity with QoL was observed for social well-being. Participants in the “high activity” cluster exhibited on average 5.4 (95%CI: 1.2 to 9.6) higher social well-being scores than participants assigned to the “inactive” cluster (p = 0.012). With regard to physical well-being a statistically significant association was observed for higher score in the “medium activity” cluster compared to the “inactive” cluster (p <0.001) with average increase of 4.2 (95%CI: 1.7 to 6.6) units. The coefficients for the “high activity” 2.4 (95%CI: -1.9 to 6.7) and “low activity” 2.1 (95%CI: -0.02 to 4.2) clusters were also positive, but did not reach statistical significance.

**Table 3:** Linear adjusted for age, sex, language region, nationality, urbanicity, participation in organized sport activities, self-reported diagnosis with at least one chronic disease, household income, parental education, and season of measurement cross-sectional association of physical activity profile cluster membership with QoL (relative to the participants in the “inactive” cluster).

Model 1 – no adjustment for established physical activity metrics				
Primary endpoint	Main predictor	Coefficient	95% CI	P-value
**Overall QoL**	Low activity	0.6	(-0.7 to 1.9)	0.366
	Medium activity	1.0	(-0.5 to 2.5)	0.197
	High activity	2.0	(-0.7 to 4.7)	0.139
**Physical well-being**	Low activity	2.1	(-0.02 to 4.2)	0.053
	Medium activity	4.2	(1.7 to 6.6)	<0.001
	High activity	2.4	(-1.9 to 6.7)	0.276
**Emotional well-being**	Low activity	0.4	(-1.4 to 2.1)	0.690
	Medium activity	0.5	(-1.5 to 2.6)	0.628
	High activity	1.6	(-2.0 to 5.2)	0.379
**Self-esteem**	Low activity	0.7	(-1.6 to 2.9)	0.571
	Medium activity	0.1	(-2.6 to 2.7)	0.968
	High activity	-0.4	(-5.1 to 4.3)	0.865
**Family connection**	Low activity	-1.1	(-3.1 to 0.9)	0.291
	Medium activity	0.2	(-2.2 to 2.6)	0.858
	High activity	0.9	(-3.3 to 5.1)	0.665
**Social well-being**	Low activity	1.1	(-0.9 to 3.2)	0.284
	Medium activity	1.7	(-0.7 to 4.1)	0.162
	High activity	5.4	(1.2 to 9.6)	0.012
**Functioning at school**	Low activity	0.5	(-1.6 to 2.6)	0.636
	Medium activity	-0.6	(-3.0 to 1.9)	0.646
	High activity	2.4	(-1.9 to 6.7)	0.269

^1^ Adjusted for age, sex, language region, nationality, urbanicity, participation in organized sport activities, self-reported diagnosis with at least one chronic disease, household income, parental education, and season of measurement

The statistically significant positive association of being in the “medium activity” cluster with the physical well-being dimension of QoL persisted after adjusting for MVPA **(Model 2, Table 4)** and SB **(Model 3, Table 5)**. While the size of the coefficient remained unaltered upon adjustment for time spent sedentary, it was attenuated from 4.2 (95%CI: 1.7 to 6.6) to 3.4 (95%CI: 0.2 to 6.6) after adjusting for MVPA. The statistically significant positive association of being assigned to the “high activity” cluster with the social well-being domain of QoL persisted only after adjustment for SB **(Model 3, Table 5)**, but was attenuated from 5.4 (95%CI: 1.2 to 9.6) to 4.8 (95%CI: 0.4 to 9.1) units. In the presence of cluster adjustments, the only statistically significant association of a conventional PA metrics with QoL was observed between MVPA and social well-being 2.4 (95%CI: 0.3 to 4.5; p = 0.025).

**Table 4:** Linear mutually adjusted^1^ cross-sectional association of physical activity profile cluster membership (relative to the participants in the inactive cluster) and MVPA (per 1h/day) with QoL.

Model 2 - additionally adjusted for MVPA							
Cluster membership					MVPA		
Primary endpoint		Coefficient	95% CI	P-value	Coefficient	95% CI	P-value
**Overall QoL**	Low activity	0.5	(-0.9 to 1.9)	0.473	0.2	(-1.1 to 1.6)	0.744
	Medium activity	0.8	(-1.2 to 2.8)	0.443			
	High activity	1.7	(-1.7 to 5.1)	0.334			
**Physical well-being**	Low activity	1.7	(-0.5 to 4.0)	0.129	0.8	(-1.3 to 2.9)	0.446
	Medium activity	3.4	(0.2 to 6.6)	0.040			
	High activity	1.1	(-4.3 to 6.5)	0.695			
**Emotional well-being**	Low activity	0.7	(-1.2 to 2.6)	0.458	-0.9	(-2.7 to 0.9)	0.314
	Medium activity	1.4	(-1.3 to 4.1)	0.307			
	High activity	3.1	(-1.5 to 7.7)	0.190			
**Self-esteem**	Low activity	1.0	(-1.5 to 3.4)	0.446	-0.7	(-3.1 to 1.6)	0.525
	Medium activity	0.8	(-2.7 to 4.3)	0.657			
	High activity	0.8	(-5.2 to 6.7)	0.798			
**Family connection**	Low activity	-1.1	(-3.3 to 1.1)	0.327	0.0	(-2.1 to 2.1)	0.999
	Medium activity	0.2	(-2.9 to 3.3)	0.893			
	High activity	0.9	(-4.4 to 6.2)	0.733			
**Social well-being**	Low activity	0.2	(-2.0 to 2.4)	0.867	2.4	(0.3 to 4.5)	0.025
	Medium activity	-0.6	(-3.8 to 2.5)	0.695			
	High activity	1.7	(-3.6 to 7.0)	0.536			
**Functioning at school**	Low activity	0.6	(-1.6 to 2.8)	0.603	-0.2	(-2.3 to 1.9)	0.826
	Medium activity	-0.3	(-3.5 to 2.9)	0.836			
	High activity	2.8	(-2.6 to 8.2)	0.314			

**Table 5:** Linear mutually adjusted^1^ cross-sectional association of physical activity profile cluster membership (relative to the participants in the inactive cluster) and sedentary behavior (per 1h/day) with QoL.

Model 3 – additionally adjusted for sedentary behavior							
Cluster membership					Sedentary behavior		
Primary endpoint		Coefficient	95% CI	P-value	Coefficient	95% CI	P-value
**Overall QoL**	Low activity	0.7	(-0.6 to 2.1)	0.288	0.2	(-0.2 to 0.6)	0.388
	Medium activity	1.2	(-0.4 to 2.8)	0.137			
	High activity	2.3	(-0.4 to 5.1)	0.101			
**Physical well-being**	Low activity	2.2	(0.01 to 4.3)	0.049	0.1	(-0.5 to 0.8)	0.711
	Medium activity	4.3	(1.8 to 6.9)	<0.001			
	High activity	2.6	(-1.8 to 7.0)	0.252			
**Emotional well-being**	Low activity	0.6	(-1.2 to 2.4)	0.524	0.3	(-0.2 to 0.9)	0.237
	Medium activity	0.9	(-1.3 to 3.0)	0.416			
	High activity	2.1	(-1.6 to 5.9)	0.258			
**Self-esteem**	Low activity	1.1	(-1.3 to 3.4)	0.374	0.6	(-0.1 to 1.3)	0.109
	Medium activity	0.7	(-2.1 to 3.5)	0.608			
	High activity	0.5	(-4.3 to 5.3)	0.836			
**Family connection**	Low activity	-1.0	(-3.1 to 1.1)	0.356	0.2	(-0.5 to 0.8)	0.605
	Medium activity	0.4	(-2.1 to 2.9)	0.746			
	High activity	1.2	(-3.1 to 5.5)	0.589			
**Social well-being**	Low activity	0.8	(-1.3 to 3.0)	0.428	-0.4	(-1.0 to 0.2)	0.218
	Medium activity	1.2	(-1.3 to 3.8)	0.332			
	High activity	4.8	(0.4 to 9.1)	0.030			
**Functioning at school**	Low activity	0.7	(-1.4 to 2.8)	0.521	0.3	(-0.3 to 0.9)	0.391
	Medium activity	-0.2	(-2.8 to 2.3)	0.853			
	High activity	2.8	(-1.5 to 7.2)	0.203			

### Predictive association

There was no evidence for an association of cluster membership at the SOPHYA1 baseline with QoL or its dimensions at SOPHYA2 when adjusting for the baseline QoL or its dimensions irrespective of adjustment for established PA metrics **(S6–S11 Tables)**.

## Discussion

The data driven unsupervised analysis of PA profiles derived from accelerometer measurements during the weekend identified clusters of youth with different behavioral patterns. The most discriminating PA characteristics between the profiles were the mean intensity of PA and the time spent in SB. A similar study clustered PA including SB based on accelerometer data obtained from pre-pubertal children in which three clusters of PA profiles were identified that primarily reflected intensity levels of PA and time spent in SB [60]. This is in contrast to results from a similar clustering approach of objectively measured PA data obtained at 5-seconds intervals from the UK Biobank. Nine distinctly different profiles were identified, but the study used data from a whole week and from more than 90’000 adults [61].

Young age and being a boy were most prevalent in the high activity profile cluster in this current study. This is in agreement with the most robust finding of a review of studies clustering PA based on a combination of conventional PA metrics [62]. It also confirms the observed objective longitudinal MVPA decline as children and adolescents grow older [63]. Furthermore, a recent study among 6- and 9-year old children confirmed the overrepresentation of boys of young age in the cross-sectional and longitudinal high activity classes. It fitted cross-sectional and longitudinal latent profile analysis models to accelerometer derived proportions of time spent in MVPA and sedentary time for weekdays and weekends. Interestingly, participants assigned to high activity profiles, in which most children achieved the recommended MVPA guidelines, were more likely to be active at weekends than on weekdays. Participation in out-of-school activities contributed importantly to changes on the patterns of PA over time [64]. The importance of the pre-school, weekend [31, 32], and holiday [65] activities is further reinforced by a study conducted among children between 7 and 11 years old to examine day-to-day PA variability. The MVPA levels were most stable during the pre-school segment of the day [66]. While differences in PA patterns between weekend days and weekdays were previously reported for the young [67, 68], it is not known whether the observed health benefit in adults reaching activity guidelines during the weekend only [69] extends to young age.

This study did not provide evidence for cross-sectional or predictive associations of either the newly derived PA cluster profiles or the conventional metrics of PA with overall QoL among children and adolescents living in Switzerland during weekend days. Overall QoL may not be the optimal endpoint for assessing the impact of PA behavior on the QoL in youth. To date, PA-related behaviors were found to be positively associated with children’s and adolescents’ psychological well-being [70, 71], social well-being [72, 73] autonomy and parents relations [74] and functioning at school [73] as dimensions of QoL. This QoL enhancement can be mediated in part by positive PA effect on the improvement of cardiorespiratory fitness [71], self-concept [75], and subjective happiness [75]. There is also empirical evidence for a negative association between SB and several QoL dimensions including psychological well-being [76], social support [76, 77], physical well-being [77], and school functioning [76] dimensions. Also in this study, some associations with QoL sub-domains were observed.

The most consistent association between PA and QoL subdomains was the observed positive association of the high activity profile cluster with the social well-being dimension, which disappeared upon MVPA, but not SB adjustment. In mutually adjusted models, MVPA was only associated with the social well-being dimension, but not any other QoL domain. PA, primarily MVPA, may therefore explain the variability of the social dimension of QoL better than the variability of other dimensions of QoL among children and adolescents. Evidence for the positive association between self-reported as well as objectively measured PA and social well-being among children and adolescents has been provided before [72, 73]. Some evidence additionally points to a causal link between the two constructs. A previous two year longitudinal study with three follow-up times was conducted among adolescents in France. The bidirectional association between PA and several dimensions of health related QoL was strongest for the social well-being dimension of health related QoL [78]. In Australia, another study based on longitudinal population-based data obtained at ages 12 and again five years later provided evidence for the predictive association between higher levels of PA and QoL, mainly driven by improvement in the physical and social well-being dimensions [79].

It is worth mentioning the positive association between activity profile clusters and physical well-being, which was stronger for the medium activity clusters than for the highest activity clusters. The associations were not sensitive for adjustment for MVPA or SB. This finding may suggest that low and medium PA levels provide the right balance between maintaining physical well-being and creating a healthy strain on the body. Light intensity PA was previously associated with higher health related QoL in girls [80].

While in adults, evidence from randomized trials points to causal short- and mid-term effects of conventional PA metrics on QoL [81, 82], very few randomized trials were conducted in children or adolescents, most of them in subgroups with specific health conditions such as cancer, type 1 diabetes, asthma, or mental health disorders. A seven-month, school-based cluster-randomized controlled PA intervention in 10-year old children in Norway did not find an effect on the overall QoL or its dimensions [83]. A randomized controlled trial in Swiss elementary school children found little effect of a school-based PA program on QoL [84].

## Strengths and limitations

The fact that the study was cohort and population-based is a major strength. This is in addition to the objective measurement of PA, which diminishes self-reporting bias in PA assessment compared to subjective PA measurement [22, 23]. Given the opportunity presented by the availability of the accelerometer-recorded weekend PA data, this study applied the k-medoid algorithm to the distance matrix of profiles obtained by pairwise alignments of PA time series using the DTW algorithm to extract clusters of PA profiles. Then, it assessed the role of PA profiles in QoL, as indicator of health, beyond conventional PA metrics, which is considered main added value of this study. The assessment of QoL was based on the KINDL® questionnaire, which is reliable and valid instrument [3].

Among the main limitations of the study is the relatively small sample size, in particular for the predictive analysis of the association of PA at baseline with QoL at the follow-up. The small sample size might have precluded identification of small or poorly separated clusters that might nevertheless capture aspects of PA variability beyond MVPA and SB that are relevant to health. This could explain the broad absence of associations between PA cluster profiles and QoL in models adjusted for established conventional PA metrics. An additional limitation of the study is the parent-proxy report on children’s and adolescents’ QoL at baseline, which compromises the essence of QoL as the individual’s subjective perception of his or her health [85]. The correlation between parent-proxy versus self-report of QoL among children and adolescents has previously been reported to be poor [86]. We acknowledge the bias in the predictive association between PA at baseline and QoL that may have been introduced due to loss to follow-up. Follow-up participants tended to be younger, more physically active and with better QoL. This may have led to an underestimation of any true association between PA and QoL.

## Conclusion

In this first population-based study that derived among children and adolescents data driven clusters of objectively measured PA profiles on the weekend no consistent and independent associations of these clusters with overall and domain-specific QoL were observed.

Because PA decreases with age and during the transition from childhood to adolescence [87, 88], PA promotion and its relevance to QoL remain important research topics. Future research based on larger longitudinal study samples with more than two follow-up time points of children and adolescents is needed to derive novel accelerometer derived PA profiles and to associate them with QoL dimensions.

## Supporting information

S1 Fig Study sample for physical activity profiles’ clustering and cross-sectional association with quality of life. (TIFF)

S2 Fig Study sample for predictive association of physical activity profiles’ clustering at baseline with quality of life at follow-up. (TIFF)

S3 Fig Physical activity pattern (counts in 15-seconds epoch) of the clusters’ two medoids (participants). (TIFF)

S4 Fig Distributions of summaries of physical activity patterns per cluster. (TIFF)

S1 Table a. Baseline characteristics of SOPHYA 1 participants compared with participants included only in the cross-sectional analysis of the study. b. Baseline characteristics of participants included in the cross-sectional analysis only compared to participants included in the predictive analysis.
(PDF)

S2 Table Characteristics of study participants at baseline (SOPHYA; 2013) by cluster of physical activity profile. (PDF)

S3 Table Linear adjusted cross-sectional association of physical activity cluster membership (relative to the participants in the lower activity cluster) with QoL. (PDF)

S4 Table Linear mutually adjusted cross-sectional association of physical activity profile cluster membership (relative to the participants in the lower activity cluster) and MVPA (per 1h/day) with QoL. (PDF)

S5 Table Linear mutually adjusted cross-sectional association of physical activity profile cluster membership (relative to the participants in the lower activity cluster) and sedentary behavior (per 1h/day) with QoL. (PDF)

S6 Table Linear adjusted predictive association of physical activity profile cluster membership (relative to the participants in the inactive cluster) at baseline with QoL at follow-up. (PDF)

S7 Table Linear mutually adjusted predictive association of physical activity profile cluster membership (relative to the participants in the inactive cluster) and MVPA (per 1h/day) at baseline with QoL at follow-up. (PDF)

S8 Table Linear mutually adjusted predictive association of physical activity profile cluster membership (relative to the participants in the inactive cluster) and sedentary behavior (per 1h/day) at baseline with QoL at follow-up. (PDF)

S9 Table Linear adjusted predictive association of physical activity profile cluster membership (relative to the participants in the lower activity cluster) at baseline with QoL at follow-up. (PDF)

S10 Table Linear mutually adjusted predictive association of physical activity profile cluster membership (relative to the participants in the lower activity cluster) and MVPA (per 1h/day) at baseline with QoL at follow-up. (PDF)

S11 Table Linear mutually adjusted predictive association of physical activity profile cluster membership (relative to the participants in the lower activity cluster) and sedentary behavior (per 1h/day) at baseline with QoL at follow-up. (PDF)

S1 Data (PDF)

S2 Data (PDF)

## Abbreviations

DTW: Dynamic Time Warping
MVPA: Moderate-to-vigorous physical activity
PA: Physical activity
QoL: Quality of life
SB: Sedentary behavior
SOPHYA: Swiss children’s Objectively measured PHYsical Activity
WHO: World Health Organization
