# Supplementary figures and images for: Weekend physical activity profiles and their relationship with quality of life: The SOPHYA cohort of Swiss children and adolescents
Source: PLoS One. 2024 May 31;19(5):e0298890. doi: 10.1371/journal.pone.0298890 (PMC11142694; doi:10.1371/journal.pone.0298890)

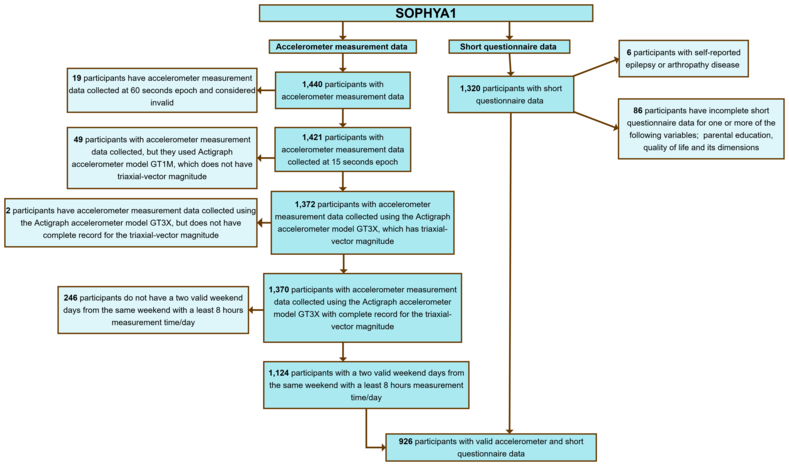

Supplement: S1 Fig — (TIFF) [file pone.0298890.s001.tiff]

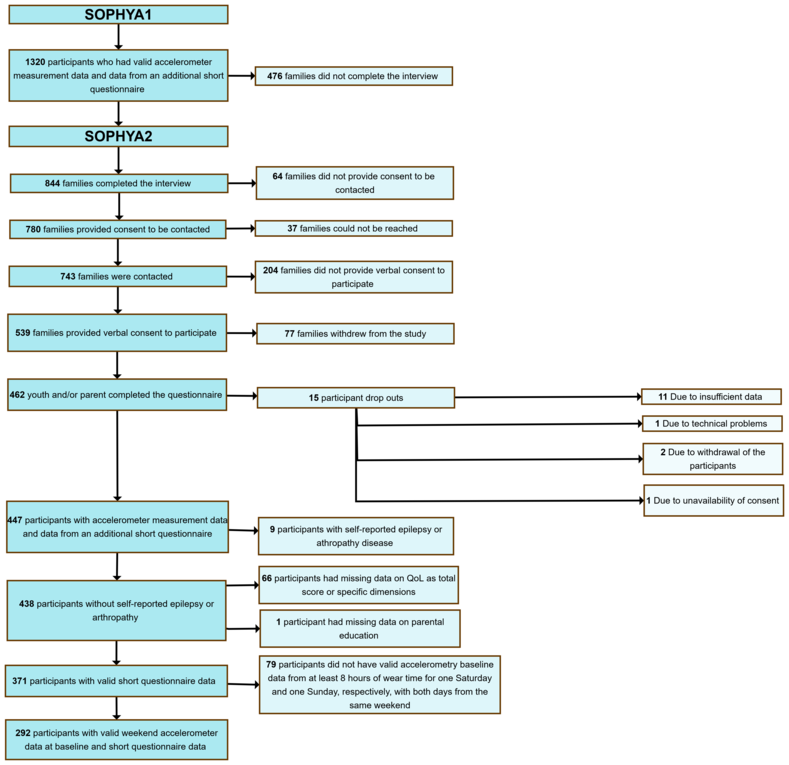

Supplement: S2 Fig — (TIFF) [file pone.0298890.s002.tiff]

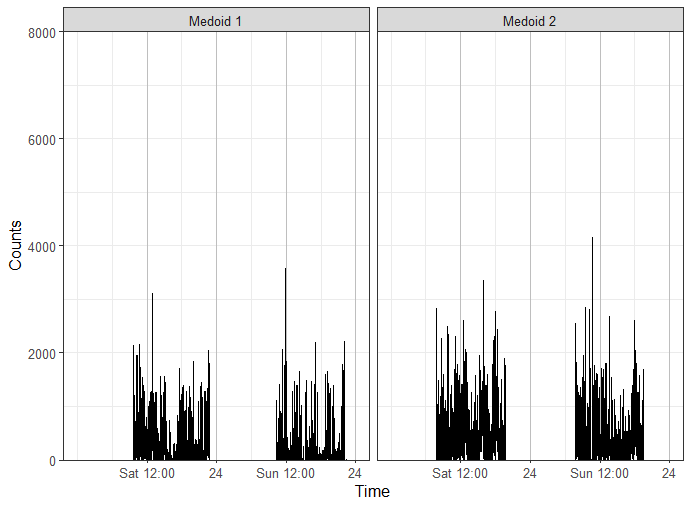

Supplement: S3 Fig — (TIFF) [file pone.0298890.s003.tiff]

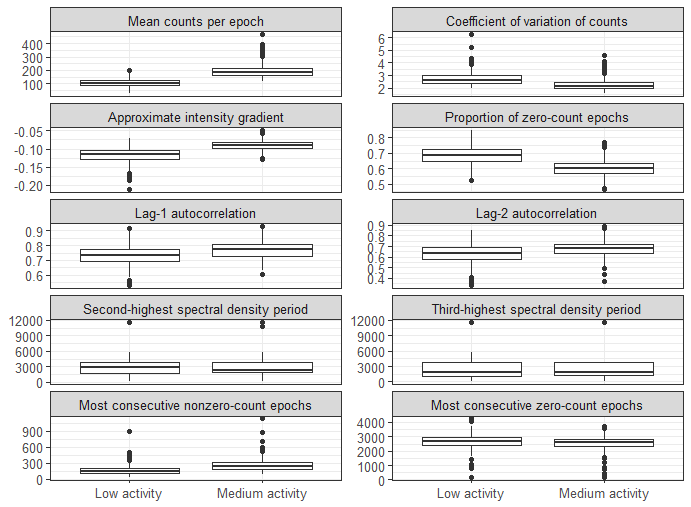

Supplement: S4 Fig — (TIFF) [file pone.0298890.s004.tiff]
