# Supplementary material for: Weekend physical activity profiles and their relationship with quality of life: The SOPHYA cohort of Swiss children and adolescents
Source: PLoS One. 2024 May 31;19(5):e0298890. doi: 10.1371/journal.pone.0298890 (PMC11142694; doi:10.1371/journal.pone.0298890)
Supplement: S1 Table — a. Baseline characteristics of SOPHYA 1 participants compared with participants included only in the cross-sectional analysis of the study. b. Baseline characteristics of participants included in the cross-sectional analysis only compared to participants included in the predictive analysis. (PDF) [file pone.0298890.s005.pdf]

S1 Table. Baseline characteristics of participants included in the cross-sectional analysis only compared to participants included in the predictive analysis

Variable	Children who participated only in SOPHYA1 N = 634 Mean (SD) / N (%)	Children who participated in SOPHYA2 N = 292 Mean (SD) / N (%)	95% CI	P-value
Socio-demographic characteristics				
**Age**	11.3 (2.6)	10.1 (2.3)	(0.9 to 1.5)	<0.001[1]
**Sex**				
- *Boy*	315.0 (49.7%)	135.0 (46.2%)	0.365[2]	
- *Girl*	319.0 (50.3%)	157.0 (53.8%)		
**Language region**				
- *German*	452.0 (71.3%)	208.0 (71.2%)	0.374[2]	
- *French*	114.0 (18.0%)	60.0 (20.5%)		
- *Italian*	68.0 (10.7%)	24.0 (8.2%)		
**Nationality**				
- *Swiss*	435.0 (68.6%)	201.0 (68.8%)	0.749[2]	
- *Foreign nationality*	68.0 (10.7%)	27.0 (9.2%)		
- *Swiss dual citizen (Swiss and foreign nationality)*	131.0 (20.7%)	64.0 (21.9%)		
**Urbanicity**				
- *Agglomeration*	298.0 (47.0%)	140.0 (47.9%)	0.582[2]	
- *Rural*	215.0 (33.9%)	90.0 (30.8%)		
- *Urban*	121.0 (19.1%)	62.0 (21.2%)		
**Parental education**[3]				
- *Apprenticeship*	291.0 (45.9%)	118.0 (40.0%)	0.102[4]	
- *High school diploma*	137.0 (21.6%)	77.0 (26.4%)		
- *Higher vocational training*	110.0 (17.4%)	58.0 (19.9%)		
- *Undefined category*	54.0 (8.5%)	30.0 (10.3%)		
- *Compulsory school*	29.0 (4.6%)	5.0 (1.7%)		
- *Diploma school*	12.0 (1.9%)	4.0 (1.4%)		
- *Not willing to provide information*	1.0 (0.2%)	0.0 (0.0%)		
**Household income**				
- *≤ 6,000 CHF*	144.0 (22.7%)	51.0 (17.5%)	0.124[1]	
- *6,001 to 9,000 CHF*	206.0 (32.5%)	93.0 (31.8%)		
- *9,000 and more CHF*	213.0 (33.6%)	121.0 (41.4%)		
- *Not willing to provide information*	21.0 (3.3%)	10.0 (3.4%)		
**Health indicators**				

**Self-reported diagnosis with at least one chronic disease**[5]				
- *Did not have any of the chronic diseases*	422.0 (66.6%)	214.0 (73.3%)	0.048[2]	
- *Had at least one chronic disease*	212.0 (33.4%)	78.0 (26.7%)		
**Quality of life**				
- *Overall QoL*	80.5 (8.6)	82.4 (7.6)	(-3.0 to -0.8)	<0.001[1]
- *Physical well-being*	83.9 (13.3)	85.1 (12.0)	(-3.0 to 0.5)	0.158[1]
- *Emotional well-being*	85.9 (10.9)	87.5 (10.2)	(-3.0 to -0.1)	0.037[1]
- *Self-esteem*	75.0 (14.3)	77.2 (12.1)	(-4.0 to -0.4)	0.017[1]
- *Family connection*	81.4 (12.7)	82.1 (12.1)	(-2.4 to 1.0)	0.422[1]
- *Social well-being*	77.9 (13.0)	79.1 (11.3)	(-2.8 to 0.5)	0.167[1]
- *Functioning at school*	79.0 (15.1)	83.5 (13.5)	(-6.4 to -2.5)	<0.001[1]
**Use of the accelerometer**				
**Season of measurement**				
- *Spring*	197.0 (31.1%)	72.0 (24.7%)	0.151[2]	
- *Summer*	83.0 (13.1%)	45.0 (15.4%)		
- *Autumn*	143.0 (22.6%)	79.0 (27.1%)		
- *Winter*	211.0 (33.3%)	96.0 (32.9%)		
**Conventional physical activity measures during the weekend**				
**Sedentary Behavior during weekend days**[6]				
- *Average time in sedentary behavior (hours/day)*	7.7 (1.6)	7.3 (1.6)	(0.2 to 0.6)	<0.001[1]
**Moderate to Vigorous Physical Activity during weekend days**[7]				
- *Average time in moderate to vigorous physical activity (hours/day)*	1.1 (0.7)	1.3 (0.7)	(-0.3 to -0.1)	<0.001[1]

[1] P-value from student's t-test
[2] P-value from the chi-squared test
[3] Highest parental education
[4] P-value from Finsher's exact test
[5] The participant self-reported at least one of the following chronic diseases: asthma, hay fever, allergy, atopic dermatitis, diabetes mellitus, chronic enteritis, hypertension, epilepsy, arthropathy and attention deficit hyperactivity disorder. Or any other chronic disease not specifically included in the mentioned list
[6] Derived by ActiLife v6.13.3, which is defined as an intensity of less than 100 cpm
[7] Derived by ActiLife v6.13.3, which is based on the age-dependent cut-offs of Freedson with a threshold of four metabolic equivalents
