# Supplementary material for: Weekend physical activity profiles and their relationship with quality of life: The SOPHYA cohort of Swiss children and adolescents
Source: PLoS One. 2024 May 31;19(5):e0298890. doi: 10.1371/journal.pone.0298890 (PMC11142694; doi:10.1371/journal.pone.0298890)
Supplement: S2 Table — (PDF) [file pone.0298890.s006.pdf]

S2 Table. Characteristics of study participants at baseline (SOPHYA; 2013) by cluster of physical activity profile

N = 926				
	**High activity n = 396 (42.8%)**	**Lower activity n = 530 (57.2%)**	**95% CI**	**P-value**
**Variable**	**Mean (SD)/ n (%)**	**Mean (SD)/ n (%)**		
**Socio-demographic characteristics**				
**Age**	9.7 (2.1)	11.8 (2.5)	(1.8 to 2.4)	<0.001[1]
**Sex**				
- *Boy*	229.0 (57.8%)	221.0 (41.7%)	<0.001[2]	
- *Girl*	167.0 (42.2%)	309.0 (58.3%)		
**Language region**				
- *German*	293.0 (74%)	367.0 (69.2%)	0.286[2]	
- *French*	67.0 (16.9%)	107.0 (20.2%)		
- *Italian*	36.0 (9.1%)	56.0 (10.6%)		
**Nationality**				
- *Swiss*	275.0 (69.4%)	361.0 (68.1%)	0.762[2]	
- *Foreign nationality*	42.0 (10.6%)	53.0 (10%)		
- *Swiss dual citizen (Swiss and foreign nationality)*	79.0 (19.9%)	116.0 (21.9%)		
**Urbanicity**				
- *Agglomeration*	176.0 (44.4%)	262.0 (49.4%)	0.310[2]	
- *Rural*	136.0 (34.3%)	169.0 (31.9%)		
- *Urban*	84.0 (21.2%)	99.0 (18.7%)		
**Parental education**				
- *Apprenticeship*	164.0 (41.4%)	245.0 (46.2%)	0.116[3]	
- *High school diploma*	83.0 (21.0%)	131.0 (24.7%)		
- *Higher vocational training*	82.0 (20.7%)	86.0 (16.2%)		
- *Undefined category*	44.0 (11.1%)	40.0 (7.5%)		
- *Compulsory school*	14.0 (3.5%)	20.0 (3.8%)		
- *Diploma school*	8.0 (2.0%)	8.0 (1.5%)		
- *Not willing to provide information*	1.0 (0.3%)	0.0 (0.0%)		
**Household income**				
- *≤ 6,000 CHF*	70.0 (17.7%)	125.0 (23.6%)	0.051[2]	
- *6,001 to 9,000 CHF*	121.0 (30.6%)	178.0 (33.6%)		
- *9,000 and more CHF*	159.0 (40.2%)	175.0 (33%)		
- *Not willing to provide information*	17.0 (4.3%)	14.0 (2.6%)		

- *Missing*	29.0 (7.3%)	38.0 (7.2%)		
**Health indicators**				
**Self-reported diagnosis with at least one chronic disease**				
- *Did not have any of the chronic diseases*	283.0 (71.5%)	353.0 (66.6%)	0.132[2]	
- *Had at least one chronic disease*	113.0 (28.5%)	177.0 (33.4%)		
**Quality of life**				
- *Overall QoL*	82.3 (7.3)	80.2 (8.9)	(-3.1 to -1.0)	<0.001[1]
- *Physical well-being*	86.7 (11.7)	82.5 (13.5)	(-5.9 to -2.6)	<0.001[1]
- *Emotional well-being*	87.0 (9.7)	86.0 (11.4)	(-2.4 to 0.3)	0.144[1]
- *Self-esteem*	76.3 (13)	75.3 (14.1)	(-2.8 to 0.7)	0.246[1]
- *Family connection*	81.4 (11.8)	81.7 (13)	(-1.3 to 1.9)	0.701[1]
- *Social well-being*	78.9 (10.9)	77.9 (13.6)	(-2.6 to 0.6)	0.214[1]
- *Functioning at school*	83.6 (13.4)	78.0 (15.2)	(-7.5 to -3.8)	<0.001[1]
**Use of the accelerometer**				
**Season of measurement**				
- *Spring*	99.0 (25%)	170.0 (32.1%)	<0.001[2]	
- *Summer*	73.0 (18.4%)	55.0 (10.4%)		
- *Autumn*	115.0 (29.0%)	107.0 (20.0%)		
- *Winter*	109.0 (27.5%)	198.0 (37.4%)		
**Conventional physical activity measures during the weekend**				
**Sedentary Behavior during weekend days on weekend days**				
- *Average time in sedentary in hours/day*	6.7 (1.3)	8.2 (1.5)	(1.3 to1.6)	<0.001[1]
**Moderate to Vigorous Physical Activity**				
- *Average time in moderate to vigorous physical activity in hours/day*	1.7 (0.6)	0.7 (0.4)	(-1.1 to -0.9)	<0.001[1]
**Mean counts per epoch on weekend days**	196.0 (46.4)	105.0 (28.4)	(-96.6 to -86.2)	<0.001[1]

[1] P-value from student's t-test
[2] P-value from the chi-squared test
[3] P-value from Fisher's exact test
