# Supplementary material for: Weekend physical activity profiles and their relationship with quality of life: The SOPHYA cohort of Swiss children and adolescents
Source: PLoS One. 2024 May 31;19(5):e0298890. doi: 10.1371/journal.pone.0298890 (PMC11142694; doi:10.1371/journal.pone.0298890)
Supplement: S3 Table — (PDF) [file pone.0298890.s007.pdf]

S3 Table. Linear adjusted[1] cross-sectional association of physical activity cluster membership (relative to the participants in the lower activity cluster) with QoL

Model 1 - no adjustment for established physical activity metrics				
**Primary endpoint**	**Main predictor**	**Coefficient**	**95% CI**	**P-value**
**Overall QoL**	High activity	0.6	(-0.6 to 1.7)	0.338
**Physical well-being**	High activity	2.1	(0.2 to 3.9)	0.029
**Emotional well-being**	High activity	0.2	(-1.4 to 1.7)	0.824
**Self-esteem**	High activity	-0.1	(-2.1 to 1.9)	0.925
**Family connection**	High activity	0.2	(-1.6 to 2.0)	0.854
**Social well-being**	High activity	1.2	(-0.6 to 3.0)	0.210
**Functioning at school**	High activity	-0.1	(-1.9 to 1.8)	0.953

[1] Adjusted for age, sex, language region, nationality, urbanicity, participation in organized sport activities, self-reported diagnosis with at least one chronic disease, household income, parental education, and season of measurement
