# Supplementary material for: Weekend physical activity profiles and their relationship with quality of life: The SOPHYA cohort of Swiss children and adolescents
Source: PLoS One. 2024 May 31;19(5):e0298890. doi: 10.1371/journal.pone.0298890 (PMC11142694; doi:10.1371/journal.pone.0298890)
Supplement: S4 Table — (PDF) [file pone.0298890.s008.pdf]

S4 Table. Linear mutually adjusted[1] cross-sectional association of physical activity profile cluster membership (relative to the participants in the lower activity cluster) and MVPA (per 1h/day) with QoL

Model 2 – additionally adjusted for MVPA							
Cluster membership					MVPA		
**Primary endpoint**		**Coefficient**	**95% CI**	**P-value**	**Coefficient**	**95% CI**	**P-value**
**Overall QoL**	High activity	0.1	(-1.3 to 1.5)	0.895	0.6	(-0.5 to 1.8)	0.278
**Physical well-being**	High activity	1.0	(-1.3 to 3.3)	0.410	1.5	(-0.3 to 3.3)	0.114
**Emotional well-being**	High activity	0.3	(-1.6 to 2.3)	0.721	-0.2	(-1.8 to 1.3)	0.762
**Self-esteem**	High activity	0.4	(-2.1 to 2.9)	0.765	-0.6	(-2.6 to 1.3)	0.526
**Family connection**	High activity	-0.1	(-2.3 to 2.1)	0.935	0.3	(-1.4 to 2.1)	0.698
**Social well-being**	High activity	-0.9	(-3.2 to 1.3)	0.409	2.8	(1.0 to 4.6)	0.002
**Functioning at school**	High activity	-0.1	(-2.3 to 2.2)	0.939	0.05	(-1.8 to 1.9)	0.961

[1] Adjusted for age, sex, language region, nationality, urbanicity, participation in organized sport activities, self-reported diagnosis with at least one chronic disease, household income, parental education, season of measurement, and additionally adjusted for MVPA
