# Supplementary material for: Weekend physical activity profiles and their relationship with quality of life: The SOPHYA cohort of Swiss children and adolescents
Source: PLoS One. 2024 May 31;19(5):e0298890. doi: 10.1371/journal.pone.0298890 (PMC11142694; doi:10.1371/journal.pone.0298890)
Supplement: S5 Table — (PDF) [file pone.0298890.s009.pdf]

S5 Table. Linear mutually adjusted[1] cross-sectional association of physical activity profile cluster membership (relative to the participants in the lower activity cluster) and sedentary behavior (per 1h/day) with QoL

Model 3 – additionally adjusted for sedentary behavior							
Cluster membership					Sedentary behavior		
**Primary endpoint**		**Coefficient**	**95% CI**	**P-value**	**Coefficient**	**95% CI**	**P-value**
**Overall QoL**	High activity	0.7	(-0.5 to 1.9)	0.285	0.1	(-0.3 to 0.5)	0.585
**Physical well-being**	High activity	2.0	(0.1 to 4.0)	0.038	-0.02	(-0.6 to 0.6)	0.949
**Emotional well-being**	High activity	0.4	(-1.2 to 2.0)	0.634	0.2	(-0.3 to 0.8)	0.339
**Self-esteem**	High activity	0.4	(-1.7 to 2.4)	0.739	0.5	(-0.1 to 1.2)	0.123
**Family connection**	High activity	0.3	(-1.6 to 2.2)	0.756	0.1	(-0.5 to 0.8)	0.625
**Social well-being**	High activity	0.7	(-1.2 to 2.6)	0.447	-0.5	(-1.1 to 0.11)	0.105
**Functioning at school**	High activity	0.1	(-1.8 to 2.1)	0.882	0.2	(-0.4 to 0.9)	0.456

[1] Adjusted for age, sex, language region, nationality, urbanicity, participation in organized sport activities, self-reported diagnosis with at least one chronic disease, household income, parental education, season of measurement, and additionally adjusted for sedentary behavior
