# Supplementary material for: Weekend physical activity profiles and their relationship with quality of life: The SOPHYA cohort of Swiss children and adolescents
Source: PLoS One. 2024 May 31;19(5):e0298890. doi: 10.1371/journal.pone.0298890 (PMC11142694; doi:10.1371/journal.pone.0298890)
Supplement: S6 Table — (PDF) [file pone.0298890.s010.pdf]

S6 Table. Linear adjusted[1] predictive association of physical activity profile cluster membership (relative to the participants in the inactive cluster) at baseline with QoL at follow-up

Model 1 – no additional adjustment for established physical activity metrics				
**Primary endpoint**	**Main predictor**	**Coefficient**	**95% CI**	**P-value**
**Overall QoL**	Low activity	1.5	(-1.6 to 4.5)	0.350
	Medium activity	0.4	(-2.9 to 3.7)	0.799
	High activity	-0.3	(-6.3 to 5.7)	0.922
**Physical well-being**	Low activity	2.7	(-1.9 to 7.2)	0.251
	Medium activity	1.4	(-3.5 to 6.4)	0.564
	High activity	3.1	(-5.7 to 11.9)	0.486
**Emotional well-being**	Low activity	0.8	(-3.1 to 4.6)	0.696
	Medium activity	-0.4	(-4.6 to 3.7)	0.846
	High activity	-3.1	(-10.6 to 4.3)	0.410
**Self-esteem**	Low activity	2.0	(-3.3 to 7.4)	0.453
	Medium activity	1.0	(-4.8 to 6.8)	0.734
	High activity	-5.8	(-16.3 to 4.6)	0.273
**Family connection**	Low activity	3.1	(-1.3 to 7.5)	0.171
	Medium activity	1.5	(-3.3 to 6.2)	0.542
	High activity	4.5	(-4.1 to 13.0)	0.307
**Social well-being**	Low activity	-1.9	(-6.2 to 2.5)	0.402
	Medium activity	0.1	(-4.6 to 4.9)	0.957
	High activity	-0.2	(-8.8 to 8.3)	0.955
**Functioning at school**	Low activity	2.5	(-3.1 to 8.1)	0.384
	Medium activity	-0.1	(-6.2 to 6.0)	0.967
	High activity	0.7	(-10.3 to 11.6)	0.903

[1] Adjusted for age, sex, language region, nationality, urbanicity, participation in organized sport activities, self-reported diagnosis with at least one chronic disease, household income, parental education, season of measurement and respective QoL domain at baseline
