# Supplementary material for: Weekend physical activity profiles and their relationship with quality of life: The SOPHYA cohort of Swiss children and adolescents
Source: PLoS One. 2024 May 31;19(5):e0298890. doi: 10.1371/journal.pone.0298890 (PMC11142694; doi:10.1371/journal.pone.0298890)
Supplement: S7 Table — (PDF) [file pone.0298890.s011.pdf]

S7 Table. Linear mutally adjusted[1] predictive association of physical activity profile cluster membership (relative to the participants in the inactive cluster) and MVPA (per 1h/day) at baseline with QoL at follow-up

Model 2 – additionally adjusted for MVPA							
Cluster membership					MVPA		
**Primary endpoint**		**Coefficient**	**95% CI**	**P-value**	**Coefficient**	**95% CI**	**P-value**
**Overall QoL**	Low activity	1.3	(-2.0 to 4.5)	0.443	0.6	(-2.2 to 3.5)	0.669
	Medium activity	-0.2	(-4.5 to 4.1)	0.938			
	High activity	-1.2	(-8.4 to 6.0)	0.751			
**Physical well-being**	Low activity	3.6	(-1.1 to 8.4)	0.135	-2.9	(-7.1 to 1.3)	0.176
	Medium activity	4.2	(-2.1 to 10.6)	0.192			
	High activity	7.1	(-3.4 to 17.7)	0.184			
**Emotional well-being**	Low activity	0.3	(-3.7 to 4.4)	0.870	1.3	(-2.3 to 4.9)	0.473
	Medium activity	-1.7	(-7.1 to 3.7)	0.543			
	High activity	-4.9	(-13.9 to 4.0)	0.279			
**Self-esteem**	Low activity	1.8	(-3.8 to 7.4)	0.527	0.7	(-4.3 to 5.7)	0.774
	Medium activity	0.3	(-7.2 to 7.8)	0.938			
	High activity	-6.8	(-19.4 to 5.7)	0284			
**Family connection**	Low activity	3.2	(-1.5 to 7.8)	0.179	-0.3	(-4.4 to 3.8)	0.901
	Medium activity	1.7	(-4.5 to 7.9)	0.584			
	High activity	4.8	(-5.5 to 15.1)	0.358			
**Social well-being**	Low activity	-1.9	(-6.5 to 2.7)	0.411	0.1	(-3.9 to 4.2)	0.946
	Medium activity	0.00	(-6.1 to 6.1)	0.999			
	High activity	-0.4	(-10.6 to 9.7)	0.933			
**Functioning at school**	Low activity	0.9	(-5.0 to 6.8)	0.759	4.8	(-0.4 to 10.0)	0.071
	Medium activity	-4.7	(-12.6 to 3.1)	0.236			
	High activity	-6.0	(-19.1 to 7.1)	0.369			

[1] Adjusted for age, sex, language region, nationality, urbanicity, participation in organized sport activities, self-reported diagnosis with at least one chronic disease, household income, parental education, season of measurement, respective QoL domain at baseline, and additionally adjusted for MVPA
