# Supplementary material for: Weekend physical activity profiles and their relationship with quality of life: The SOPHYA cohort of Swiss children and adolescents
Source: PLoS One. 2024 May 31;19(5):e0298890. doi: 10.1371/journal.pone.0298890 (PMC11142694; doi:10.1371/journal.pone.0298890)
Supplement: S8 Table — (PDF) [file pone.0298890.s012.pdf]

S8 Table. Linear mutually adjusted[1] predictive association of physical activity profile cluster membership (relative to the participants in the inactive cluster) and sedentary behavior (per 1h/day) at baseline with QoL at follow-up

Model 3 - additionally adjusted for sedentary behavior							
Cluster membership					Sedentary behavior		
**Primary endpoint**		**Coefficient**	**95% CI**	**P-value**	**Coefficient**	**95% CI**	**P-value**
**Overall QoL**	Low activity	1.8	(-1.4 to 5.1)	0.256	0.4	(-0.5 to 1.4)	0.398
	Medium activity	1.0	(-2.6 to 4.6)	0.581			
	High activity	0.5	(-5.8 to 6.7)	0.886			
**Physical well-being**	Low activity	2.9	(-1.9 to 7.6)	0.239	0.2	(-1.2 to 1.6)	0.779
	Medium activity	1.7	(-3.6 to 7.0)	0.523			
	High activity	3.5	(-5.7 to 12.7)	0.456			
**Emotional well-being**	Low activity	1.2	(-2.8 to 5.2)	0.565	0.4	(-0.8 to 1.6)	0.479
	Medium activity	0.2	(-4.3 to 4.7)	0.933			
	High activity	-2.3	(-10.2 to 5.5)	0.556			
**Self-esteem**	Low activity	2.0	(-3.6 to 7.6)	0.477	0.0	(-1.7 to 1.6)	0.978
	Medium activity	1.0	(-5.3 to 7.2)	0.760			
	High activity	-5.9	(-16.8 to 5.0)	0.290			
**Family connection**	Low activity	3.3	(-1.3 to 7.9)	0.157	0.3	(-1.1 to 1.6)	0.711
	Medium activity	1.8	(-3.3 to 7.0)	0.482			
	High activity	4.9	(-4.0 to 13.9)	0.279			
**Social well-being**	Low activity	-0.3	(-4.8 to 4.2)	0.903	1.7	(0.4 to 3.1)	0.013
	Medium activity	2.4	(-2.6 to 7.5)	0.338			
	High activity	2.8	(-6.0 to 11.6)	0.535			
**Functioning at school**	Low activity	2.3	(-3.6 to 8.2)	0.440	-0.2	(-2.0 to 1.6)	0.829
	Medium activity	-0.4	(-7.0 to 6.2)	0.905			
	High activity	0.3	(-11.1 to 11.8)	0.956			

[1] Adjusted for age, sex, language region, nationality, urbanicity, participation in organized sport activities, self-reported diagnosis with at least one chronic disease, household income, parental education, season of measurement, respective QoL domain at baseline, and additionally adjusted for sedentary behavior
