# Supplementary material for: Weekend physical activity profiles and their relationship with quality of life: The SOPHYA cohort of Swiss children and adolescents
Source: PLoS One. 2024 May 31;19(5):e0298890. doi: 10.1371/journal.pone.0298890 (PMC11142694; doi:10.1371/journal.pone.0298890)
Supplement: S9 Table — (PDF) [file pone.0298890.s013.pdf]

S9 Table. Linear adjusted[1] predictive association of physical activity profile cluster membership (relative to the participants in the lower activity cluster) at baseline with QoL at follow-up

Model 1 – no adjustment for established physical activity metrics				
**Primary endpoint**	**Main predictor**	**Coefficient**	**95% CI**	**P-value**
**Overall QoL**	High activity	0.4	(-2.2 to 3.0)	0.766
**Physical well-being**	High activity	1.1	(-2.7 to 4.9)	0.568
**Emotional well-being**	High activity	0.3	(-3.0 to 3.6)	0.854
**Self-esteem**	High activity	1.9	(-2.7 to 6.4)	0.420
**Family connection**	High activity	2.1	(-1.6 to 5.9)	0.261
**Social well-being**	High activity	-1.9	(-5.7 to 1.8)	0.301
**Functioning at school**	High activity	-0.7	(-5.5 to 4.1)	0.773

[1] Adjusted for age, sex, language region, nationality, urbanicity, participation in organized sport activities, self-reported diagnosis with at least one chronic disease, household income, parental education, season of measurement and respective QoL domain at baseline
