# Supplementary material for: Weekend physical activity profiles and their relationship with quality of life: The SOPHYA cohort of Swiss children and adolescents
Source: PLoS One. 2024 May 31;19(5):e0298890. doi: 10.1371/journal.pone.0298890 (PMC11142694; doi:10.1371/journal.pone.0298890)
Supplement: S10 Table — (PDF) [file pone.0298890.s014.pdf]

S10 Table. Linear mutually adjusted[1] predictive association of physical activity profile cluster membership (relative to the participants in the lower activity cluster) and MVPA (per 1h/day) at baseline with QoL at follow-up

Model 2 – additionally adjusted for MVPA							
Cluster membership					MVPA		
**Primary endpoint**		**Coefficient**	**95% CI**	**P-value**	**Coefficient**	**95% CI**	**P-value**
**Overall QoL**	High activity	0.4	(-2.9 to 3.6)	0.826	0.0	(-2.5 to 2.6)	0.973
**Physical well-being**	High activity	2.8	(-1.9 to 7.6)	0.238	-2.3	(-6.0 to 1.4)	0.221
**Emotional well-being**	High activity	0.4	(-3.6 to 4.5)	0.825	-0.2	(-3.4 to 3.0)	0.902
**Self-esteem**	High activity	3.2	(-2.4 to 8.8)	0.269	-1.7	(-6.1 to 2.7)	0.440
**Family connection**	High activity	2.7	(-1.9 to 7.4)	0.245	-0.8	(-4.4 to 2.8)	0.665
**Social well-being**	High activity	-3.4	(-7.9 to 1.2)	0.145	1.9	(-1.7 to 5.6)	0.287
**Functioning at school**	High activity	-3.4	(-9.3 to 2.4)	0.249	3.7	(-0.9 to 8.3)	0.118

[1] Adjusted for age, sex, language region, nationality, urbanicity, participation in organized sport activities, self-reported diagnosis with at least one chronic disease, household income, parental education, season of measurement, respective QoL domain at baseline, and additionally adjusted for MVPA
