# Supplementary material for: Weekend physical activity profiles and their relationship with quality of life: The SOPHYA cohort of Swiss children and adolescents
Source: PLoS One. 2024 May 31;19(5):e0298890. doi: 10.1371/journal.pone.0298890 (PMC11142694; doi:10.1371/journal.pone.0298890)
Supplement: S11 Table — (PDF) [file pone.0298890.s015.pdf]

S11 Table. Linear mutually adjusted[1] predictive association of physical activity profile cluster membership (relative to the participants in the lower activity cluster) and sedentary behavior (per 1h/day) at baseline with QoL at follow-up

**Model 3 – additionally adjusted for sedentary behavior**							
**Cluster membership**					**Sedentary behavior**		
**Primary endpoint**		**Coefficient**	**95% CI**	**P-value**	**Coefficient**	**95% CI**	**P-value**
**Overall QoL**	High activity	0.7	(-2.0 to 3.5)	0.600	0.4	(-0.5 to 1.3)	0.427
**Physical well-being**	High activity	1.2	(-2.9 to 5.2)	0.567	0.0	(-1.3 to 1.4)	0.922
**Emotional well-being**	High activity	0.8	(-2.6 to 4.2)	0.649	0.5	(-0.6 to 1.7)	0.360
**Self-esteem**	High activity	2.1	(-2.7 to 6.8)	0.395	0.2	(-1.4 to 1.8)	0.787
**Family connection**	High activity	2.3	(-1.6 to 6.2)	0.248	0.2	(-1.1 to 1.5)	0.778
**Social well-being**	High activity	-0.7	(-4.6 to 3.1)	0.708	1.4	(0.1 to 2.7)	0.344
**Functioning at school**	High activity	-1.0	(-6.0 to 4.0)	0.701	-0.3	(-2.0 to 1.4)	0.718

[1] Adjusted for age, sex, language region, nationality, urbanicity, participation in organized sport activities, self-reported diagnosis with at least one chronic disease, household income, parental education, season of measurement, respective QoL domain at baseline, and additionally adjusted for sedentary behavior
