# Supplementary material for: Weekend physical activity profiles and their relationship with quality of life: The SOPHYA cohort of Swiss children and adolescents
Source: PLoS One. 2024 May 31;19(5):e0298890. doi: 10.1371/journal.pone.0298890 (PMC11142694; doi:10.1371/journal.pone.0298890)
Supplement: S1 Data — (PDF) [file pone.0298890.s016.pdf]

S1a Table. Baseline characteristics of SOPHYA 1 participants compared with participants included only in the cross-sectional analysis of the study

	**Children who participated in SOPHYA1 N = 1320**	**Children who participated in the cross-sectional analysis of the study N = 926**	**P-value**
**Variable**	**Mean (SD) / N (%)**	**Mean (SD) / N (%)**	
Socio-demographic characteristics			
**Age**	11.1 (2.6)	10.9 (2.5)	0.181[1]
**Sex**			
- *Boy*	662.0 (50.2%)	450.0 (48.6%)	0.495[2]
- *Girl*	658.0 (49.8%)	476.0 (51.4%)	
**Language region**			
- *German*	915.0 (69.3%)	660.0 (71.3%)	0.515[2]
- *French*	256.0 (19.4%)	174.0 (18.8%)	
- *Italian*	149.0 (11.3%)	92.0 (9.9%)	
**Nationality**			
- *Swiss*	900.0 (68.2%)	636.0 (68.7%)	0.886[2]
- *Foreign nationality*	144.0 (10.9%)	95.0 (10.3%)	
- *Swiss dual citizen (Swiss and foreign nationality)*	276.0 (20.9%)	195.0 (21.1%)	
**Urbanicity**			
- *Agglomeration*	636.0 (48.2%)	438.0 (47.3%)	0.911[2]
- *Rural*	430.0 (32.6%)	305.0 (32.9%)	
- *Urban*	254.0 (19.2%)	183.0 (19.8%)	
**Parental education**[3]			
- *Apprenticeship*	593.0 (44.9%)	409.0 (44.2%)	0.997[4]
- *High school diploma*	297.0 (22.5%)	214.0 (23.1%)	
- *Higher vocational training*	233.0 (17.7%)	168.0 (18.1%)	
- *Undefined category*	114.0 (8.6%)	84.0 (9.1%)	
- *Compulsory school*	51.0 (3.9%)	34.0 (3.7%)	
- *Diploma school*	25.0 (1.9%)	16.0 (1.7%)	
- *Not willing to provide information*	2.0 (0.2%)	1.0 (0.1%)	
- *Missing*	5.0 (0.4%)	-	
**Household income**			
- *≤ 6,000 CHF*	278.0 (21.1%)	195.0 (21.1%)	<0.001[1]
- *6,001 to 9,000 CHF*	416.0 (31.5%)	299.0 (32.3%)	
- *9,000 and more CHF*	475.0 (36.0%)	334.0 (36.1%)	

- *Not willing to provide information*	52.0 (3.9%)	31.0 (3.3%)	
- *Missing*	99.0 (7.5%)	67.0 (7.2%)	
**Health indicators**			
**Self-reported diagnosis with at least one chronic disease**[5]			
- *Did not have any of the chronic diseases*	897.0 (68.0%)	636.0 (68.7%)	0.750[2]
- *Had at least one chronic disease*	423.0 (32.0%)	290.0 (31.3%)	
**Quality of life**			
- *Overall QoL*	80.6 (8.8)	81.1 (8.3)	0.193[1]
- *Physical well-being*	83.8 (13.9)	84.3 (12.9)	0.411[1]
- *Emotional well-being*	86 (11.5)	86.4 (10.7)	0.372[1]
- *Self-esteem*	75.2 (14.2)	75.7 (13.7)	0.359[1]
- *Family connection*	81.4 (12.8)	81.6 (12.5)	0.672[1]
- *Social well-being*	78.2 (12.9)	78.3 (12.5)	0.889[1]
- *Functioning at school*	79.7 (15.3)	80.4 (14.7)	0.271[1]
**Use of the accelerometer**			
**Weartime**			
- *Average scored time per day (minutes)*	794.0 (55.5)	798.6 (52.1)	0.072[1]
**Season of measurement**			
- *Spring*	382.0 (28.9%)	269.0 (29.0%)	0.982[2]
- *Summer*	182.0 (13.8%)	128.0 (13.8%)	
- *Autumn*	326.0 (24.7%)	222.0 (24%)	
- *Winter*	430.0 (32.6%)	307.0 (33.2%)	
**Conventional physical activity measures during the weekend**			
**Sedentary Behavior during weekend days**[6]			
- *Average time in sedentary behavior (hours/day)*	7.5 (1.7)	7.6 (1.6)	0.541[1]
**Moderate to Vigorous Physical Activity during weekend days**[7]			
- *Average time in moderate to vigorous physical activity (hours/day)*	1.1 (0.7)	1.1 (0.7)	0.158[1]

[1] P-value from student's t-test
[2] P-value from the chi-squared test
[3] Highest parental education
[4] P-value from Finsher's exact test
[5] The participant self-reported at least one of the following chronic diseases: asthma, hay fever, allergy, atopic dermatitis, diabetes mellitus, chronic enteritis, hypertension, epilepsy, arthropathy and attention deficit hyperactivity disorder. Or any other chronic disease not specifically included in the mentioned list
[6] Derived by ActiLife v6.13.3, which is defined as an intensity of less than 100 cpm
[7] Derived by ActiLife v6.13.3, which is based on the age-dependent cut-offs of Freedson with a threshold of four metabolic equivalents
